# The CC chemokine ligand (CCL) 1, upregulated by the viral transactivator Tax, can be downregulated by minocycline: possible implications for long-term treatment of HTLV-1-associated myelopathy/tropical spastic paraparesis

**DOI:** 10.1186/s12985-017-0902-6

**Published:** 2017-12-04

**Authors:** Mineki Saito, Hiroe Sejima, Tadasuke Naito, Hiroshi Ushirogawa, Toshio Matsuzaki, Eiji Matsuura, Yuetsu Tanaka, Tatsufumi Nakamura, Hiroshi Takashima

**Affiliations:** 1 Department of Microbiology, Kawasaki Medical School, 577 Matsushima, Okayama, 701-0192 Japan; 2 Department of Neurology and Geriatrics, Kagoshima University Graduate School of Medical and Dental Sciences, 8-35-1 Sakuragaoka, Kagoshima, 890-8520 Japan; 3 Department of Immunology, Graduate School of Medicine, University of the Ryukyus, 207 Uehara, Okinawa, 903-0215 Japan; 4 Department of Social Work, Faculty of Human and Social Studies, Nagasaki International University, 2825-7 Huis Ten Bosch Machi, Sasebo, Nagasaki, 859-3298 Japan

**Keywords:** HTLV-1, Tax, CCL1, HAM/TSP, Minocycline

## Abstract

**Background:**

Chemokine (C-C motif) ligand 1 (CCL1) is produced by activated monocytes/ macrophages and T-lymphocytes, and acts as a potent attractant for Th2 cells and a subset of T-regulatory (Treg) cells. Previous reports have indicated that CCL1 is overexpressed in adult T-cell leukemia cells, mediating an autocrine anti-apoptotic loop. Because CCL1 is also known as a potent chemoattractant that plays a major role in inflammatory processes, we investigated the role of CCL1 in the pathogenesis of human T-cell leukemia virus type 1 (HTLV-1)-associated myelopathy/tropical spastic paraparesis (HAM/TSP).

**Results:**

The results showed that: (1) CCL1 was preferentially expressed in HAM/TSP-derived HTLV-1-infected T-cell lines, (2) CCL1 expression was induced along with Tax expression in the Tax-inducible T-cell line JPX9, (3) transient Tax expression in an HTLV-1-negative T-cell line activated the *CCL1* gene promoter, (4) plasma levels of CCL1 were significantly higher in patients with HAM/TSP than in HTLV-1-seronegative patients with multiple sclerosis and HTLV-1-infected asymptomatic healthy carriers, and (5) minocycline inhibited the production of CCL1 in HTLV-1-infected T-cell lines.

**Conclusions:**

The present results suggest that elevated CCL1 levels may be associated with the pathogenesis of HAM/TSP. Although further studies are required to determine the in vivo significance, minocycline may be considered as a potential candidate for the long-term treatment of HAM/TSP via its anti-inflammatory effects, which includes the inhibition of CCL1 expression.

## Background

Human T-cell leukemia virus type 1 (HTLV-1) was the first human oncogenic retrovirus to be identified and associated with distinct human diseases, such as adult T-cell leukemia (ATL) [1, 2] and HTLV-1-associated myelopathy/tropical spastic paraparesis (HAM/TSP) [3, 4]. HAM/TSP is a chronic inflammatory disease of the central nervous system (CNS), pathologically characterized by perivascular lymphocytic cuffing and parenchymal lymphocytic infiltration, including HTLV-1-infected CD4^+^ T-cells [5]. This pathological condition causes severe dysfunction of the corticospinal (pyramidal) tracts of the spinal cord, resulting in urinary failures and leg paralysis, which are the main clinical symptoms of HAM/TSP [6]. In addition to these neurological symptoms, some patients with HAM/TSP also exhibit autoimmune-like inflammatory disorders such as uveitis, arthritis, T-lymphocyte alveolitis, polymyositis, and Sjögren’s syndrome [7]. Like many autoimmune diseases, pro-inflammatory cytokines, chemokines, and matrix metalloproteinases act as important effectors of tissue damage in patients with HAM/TSP [8]. Particularly, the overexpression of interferon (IFN)-stimulated genes in circulating leukocytes has been observed, and the expression correlated with the clinical severity of HAM/TSP [9]. Furthermore, increased concentrations of inflammatory markers, such as neopterin [10], tumor necrosis factor (TNF)-α, interleukin (IL)-6, and IFN-γ [11], and an increase in HTLV-1 antigen-specific intrathecal antibody synthesis [12] have been observed in the cerebrospinal fluid (CSF) of patients with HAM/TSP. These findings indicate that a pro-inflammatory environment, associated with increased numbers of HTLV-1-infected and activated T-cells, is a characteristic immunologic profile of HAM/TSP. Accordingly, anti-inflammatory drugs, such as prednisolone [13] and IFN-α [14], have been approved for the treatment of HAM/TSP. However, such drugs often have insufficient effects, various side effects, and are expensive for long-term treatment. Therefore, it is important to develop strategies that are effective and tolerable, even for long-term or lifelong treatment.

Chemokine (C-C motif) ligand 1 (CCL1) is a small glycoprotein secreted by monocytes, activated macrophages, and T-lymphocytes that belongs to a family of inflammatory cytokines known as chemokines [15, 16]. It has been reported that CCL1 is overexpressed in ATL cells, mediating an autocrine anti-apoptotic loop, along with its receptor, CC chemokine receptor 8 (CCR8), for in vivo growth and survival of leukemic cells [17]. Because CCL1 is known to induce cytokine secretion and direct the trafficking of immune cells, increased CCL1 expression might be associated with leukocyte mobilization in vivo, and thus also contributing to chronic inflammatory conditions. If this is the case, interfering with CCL1 function may be a promising approach for novel anti-inflammatory treatment against HAM/TSP.

In this study, we investigated the possible role of CCL1 in patients with HAM/TSP, and suggest minocycline as a novel immunomodulatory drug against HAM/TSP, partly due to its inhibitory effect on CCL1 expression.

## Methods

### Patients and preparation of clinical samples

This study was approved by the Research Ethics Committee of Kawasaki Medical School (approval number: 1422-3). Written informed consent was obtained from all individuals. Plasma samples from 31 patients with HAM/TSP, 13 HTLV-1-seronegative patients with multiple sclerosis (MS), 19 HTLV-1-infected asymptomatic healthy carriers (HCs), and 10 HTLV-1-uninfected normal control subjects (NCs) were analyzed. The diagnosis of HAM/TSP and MS were made according to the World Health Organization diagnostic criteria [18] and the McDonald criteria [19], respectively. The patients included in this study fulfilled the following criteria: (1) neurologically stable within 30 days before participating in this study on the condition that the current medication for each patient was kept constant, (2) for MS patients, an Expanded Disability Status Scale (EDSS) score between 3 to 7, ranging from moderate disability to essentially wheelchair-bound, (3) for HAM/TSP patients, an Osame’s Motor Disability Score (OMDS) between 4 to 9, ranging from moderate disability to essentially wheelchair-bound. Fresh peripheral blood mononuclear cells (PBMCs) were isolated using Histopaque-1077 (Sigma, St. Louis, MO, USA) density gradient centrifugation, washed twice in RPMI medium, and stored in liquid nitrogen as stocked lymphocytes until use. Plasma samples were collected before starting the therapy and stored at −80 °C until further use.

### Cell culture

Twelve HTLV-1-infected human T-cell lines (MT2, MT4, C5MJ, MT1, ATL43Tb, ATL55T, ED, TLOm1, ILT-M1, HCT1, HCT4, and HCT5) and three HTLV-1-uninfected T-cell lines (Jurkat, CEM, and Molt4) were used in this study. MT2, MT4, and C5MJ are chronically HTLV-1-infected cell lines derived from cord blood mononuclear cells exposed to HTLV-1 from patients with leukemia (i.e., HTLV-1-transformed T-cell lines). MT1, ATL43Tb, ATL55T, ED, and TLOm1 are HTLV-1-infected cell lines derived from patients with ATL (i.e., ATL cell lines). Among these ATL cell lines, only ATL55T is IL-2-dependent. ILT-M1, HCT1, HCT4, and HCT5 are IL-2-dependent HTLV-1-infected T-cell lines derived from patients with HAM/TSP. The Tax-inducible JPX9 cell line was derived from the Jurkat HTLV-1-negative human T-cell leukemia cell line and expresses biologically active Tax protein under the control of the metallothionein promoter [20]. These cells were cultured in RPMI 1640 medium supplemented with 10% heat-inactivated fetal calf serum (FCS), 50 U/ml penicillin, and 50 μg/ml streptomycin (Wako Pure Chemical, Osaka, Japan) at 37 °C and 5% CO_2_. For IL-2-dependent cell lines, 10 U/ml (for ATL55T, HCT1, HCT4, and HCT5) or 30 U/ml (for ILT-M1) of recombinant human IL-2 (Wako) were added to the culture.

### Flow cytometry

After thawing, cells were washed thrice with phosphate-buffered saline (PBS) and fixed in PBS containing 2% paraformaldehyde (Sigma, Tokyo, Japan) for 20 min at 4 °C. Fixed cells were washed with PBS containing 7% normal goat serum (Sigma) and then incubated for 15 min at room temperature with phycoerythrin-cyanin 5.1 (PC5)-labeled anti-CD4 (13B8.2) (BioLegend, San Diego, CA, USA). Isotype-matched mouse immunoglobulins were used as a control. For intracellular staining, fixed cells were washed with PBS containing 7% normal goat serum (Sigma) and permeabilized with 0.2% saponin (Sigma) in PBS and 7% normal goat serum (PBS-SAPO) for 10 min at room temperature. Permeabilized cells were washed twice and resuspended in PBS-SAPO containing fluorescein isothiocyanate (FITC)-labeled anti-Tax mAb (Lt-4). After staining for Tax, cells were stained with anti-CCL1 mAb (clone 35,305; R&D Systems, Minneapolis, Minnesota) for 20 min at room temperature. Cells were washed twice and resuspended in PBS and 7% normal goat serum containing PE-labeled goat F(ab’)2 anti-mouse IgG1 (Southern Biotechnology, Birmingham, AL, USA) for 20 min at room temperature. Finally, cells were washed twice and analyzed by standard flow cytometry using FACSCalibur and the Cell Quest software (Becton Dickinson, BD, Franklin Lakes, NJ, USA).

### Elisa

Human CCL1 was measured by ELISA using the Quantikine Human I-309/CCL1 Immunoassay kit (R&D Systems) according to the manufacturer’s instruction.

### Genomic DNA and RNA extraction and cDNA synthesis

Genomic DNA was extracted from PBMCs using the QIAamp Blood Kit (QIAGEN, Tokyo, Japan). RNA was extracted from PBMCs using the RNeasy Mini Kit with on-column DNase digestion (QIAGEN). The cDNA was synthesized with the PrimeScript® RT Reagent Kit (Takara, Kyoto, Japan). All reaction procedures were performed as suggested by the manufacturers.

### Real-time PCR analysis

To examine the HTLV-1 proviral load (PVL), quantitative PCR was performed using 100 ng of genomic DNA (roughly equivalent to 10^4^ cells) from PBMCs as previously reported [21]. The amount of HTLV-1 proviral DNA was determined using the following formula: copy number of HTLV-1 *tax* per 1 × 10^4^ PBMCs = [(copy number of tax)/(copy number of β − actin/2)] × 10^4^. All samples were examined in triplicate. TaqMan® real-time RT-PCR assays were performed to quantify the differences in the expression of *tax* and *HBZ* mRNA as previously reported [22]. *Tax*, *HBZ*, and *CCL1* mRNA expression levels were normalized to the expression of human hypoxanthine phosphoribosyltransferase 1 (*HPRT1*) (Human HPRT1 Endogenous Control 4,333,768; Applied Biosystems, Foster City, CA, USA). Human *CCL1* gene-specific assays (Applied Biosystems Hs00171072 m1) were used for *CCL1* quantification. All assays were performed in triplicate.

### Plasmids

The following pGL3-based plasmids were constructed for luciferase reporter gene assays. To amplify the *CCL1* gene promoter region harboring nucleotides from positions −1541 to +60 (where the transcription start site is set to be +1), −401 to +60, −281 to +60, and −221 to +60, PCR was carried out using the appropriate primer sets with restriction sites (Table 1) and genomic DNA derived from Jurkat cells as the template. PCR products were then digested with *Xho*I and *Hind*III, and cloned into the *Xho*I- and *Hind*III-digested pGL3-Basic Vector (Promega Corporation, Madison, WI, USA). The expression vector pCG-Tax and the control vector pCG-BL were kindly provided by Dr. J. Fujisawa (Kansai Medical University, Osaka, Japan).

**Table 1:** Primer sequences for reporter plasmid construction

Primer name	Direction	Sequence
-1541 CCL1p-F1	Forward	CAT **CTC GAG** TAT TTC ACC ACA TGT GCA TGG
-401 CCL1p-F2	Forward	CAT **CTC GAG** TGG CTG GTA TTC GTG GTG GTG
-281 CCL1p-F3	Forward	CAT **CTC GAG** GAA AAG TAA GTG AGA GGA AGT
-221 CCL1p-F4	Forward	CAT **CTC GAG** CAA GTA CTA CTG CTT CAG GGC
CCL1p-R	Reverse	CAT **AAG CTT** GGC TAA CCA AGG ACC TAG AAC

CTC GAG and AAG CTT (bold letters) are the respective restriction sites for *Xho*I and *Hind*III

### Luciferase assay

To compare the relative promoter activity, the CD4+ T-cell leukemia cell line Jurkat was used for dual luciferase assays. Jurkat cells were seeded on 12-well plates at 2.0 × 10^5^ cells per well, and were transfected with 1 μg of the reporter plasmid and 30 ng of the *Renilla* luciferase control plasmid phRL-TK, with or without 1 μg of the Tax expression plasmid pCG-Tax, using Lipofectamine LTX with PLUS reagent (Invitrogen, Carlsbad, CA, USA). *Firefly* and *Renilla* dual luciferase assays were performed 48 h post-transfection as described previously [23]. Each experiment was performed in triplicate, and the data are presented as the mean ± SD of three independent experiments, each normalized to *Renilla* activity.

### Statistical analysis

To test for significant differences among the four different groups of subjects, i.e., HAM/TSP, HTLV-1-seronegative MS, HCs, or NCs, the Kruskal-Wallis test was employed. For multiple comparisons, we used Sheffe’s F to analyze statistical differences. Correlations between variables were examined using Spearman’s rank correlation analyses. The results are presented as the mean ± SD where applicable. Values of *p* < 0.05 were considered statistically significant.

## Results

### Preferential expression of CCL1 in HTLV-1-infected T-cell lines derived from patients with HAM/TSP

**Fig. 1:**
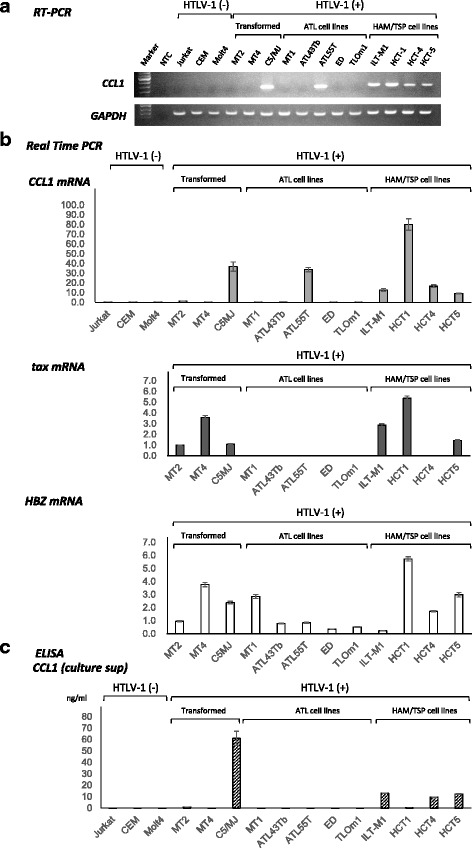
Preferential expression of CCL1 in Human T-cell leukemia virus type-1 (HTLV-1)-infected T-cell lines derived from patients with HTLV-1-associated myelopathy/tropical spastic paraparesis (HAM/TSP). **a**. Expression of *CCL1* was examined by RT-PCR in HTLV-1-infected and -uninfected T-cell lines. *CCL1* mRNA was preferentially expressed in HTLV-1-infected human T-cell lines derived from patients with HAM/TSP (4 out of 4 tested), compared with HTLV-1-transformed T-cell lines (1 out of 3) and adult T-cell leukemia (ATL) cell lines (1 out of 4). **b**. The expressions of *CCL1*, *tax*, and *HBZ* were examined by real time PCR in HTLV-1-infected and -uninfected T-cell lines. *CCL1* mRNA was preferentially expressed in HTLV-1-infected human T-cell lines derived from patients with HAM/TSP. The expression levels of the viral RNAs *tax* and *HBZ* were relatively high in T-cell lines derived from patients with HAM/TSP and HTLV-1-transformed T-cell lines when compared with those in ATL cell lines. **c**. CCL1 levels in culture supernatants from HTLV-1-infected and -uninfected human T-cell lines were assessed by ELISA. Supernatants were harvested when cells reached subconfluency. Significant levels of CCL1 was observed in the culture supernatants from HTLV-1-infected human T-cell lines derived from patients with HAM/TSP, whereas it was not detectable in any of the other cell lines tested except for the HTLV-1-transformed C5MJ cell line

First, we assessed the levels of CCL1 mRNA and protein expression in HTLV-1-infected and -uninfected human T-cell lines. As shown in Fig. 1a and b, *CCL1* mRNA was preferentially expressed in HTLV-1-infected human T-cell lines derived from patients with HAM/TSP (4 out of 4 tested), compared with HTLV-1-transformed T-cell lines (1 out of 3) and ATL cell lines (1 out of 4). Real-time PCR analysis revealed that the expression levels of the viral RNAs *tax* and *HBZ* in these HAM/TSP-derived cell lines were relatively high, in comparison with the levels in ATL cell lines (Fig. 1b). We also examined CCL1 levels in culture supernatants from HTLV-1-infected and -uninfected human T-cell lines by ELISA. As shown in Fig. 1c, significant CCL1 expression was observed in HTLV-1-infected human T-cell lines derived from patients with HAM/TSP, whereas expression was not detectable in any of the other cell lines tested except for the HTLV-1-transformed C5MJ cell line. To exclude the possibility that IL-2 in the culture medium can induce the expression of CCL1 independently of the transactivation properties of the Tax protein, we incubated HTLV-1-uninfected cell lines (Jurkat, CEM and Molt4) with 10 U/ml of IL-2 to evaluate whether the levels of CCL1 expression on the cell surface as well as the secretion of CCL1 into the culture supernatant would be affected. As a result, the incubation with IL-2 did not appreciably affect the levels of CCL1 expression (data not shown).

### Tax-dependent constitutive expression of CCL1 in tax-inducible JPX9 cells

**Fig. 2:**
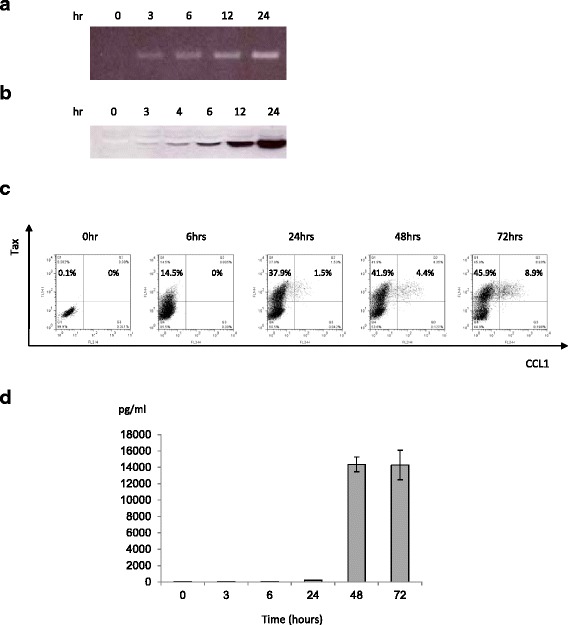
Tax-dependent constitutive expression of CCL1 in Tax-inducible JPX9 cells. RNA and protein expression of CCL1 in JPX9 cells. JPX9 is a Jurkat (HTLV-1-negative human T-cell leukemia cell line) subclone generated by stable transfection of a functional Tax expression-plasmid vector. Tax expression was induced by adding CdCl_2_ to the culture medium (final concentration: 10 μM). RT-PCR (**a**) and western blotting (**b**) showed that treatment of JPX9 cells with CdCl_2_ induced the expression of Tax at the RNA and protein levels, respectively. **c**. Flow cytometry data showed that treatment of JPX9 cells with CdCl_2_ induced CCL1 expression and that CCL1 was expressed exclusively in cells that also expressed Tax. **d**. ELISA analysis of culture supernatants showed that the addition of CdCl_2_ to the JPX9 cell culture resulted in an increase in CCL1 expression within 24 h. CCL1 levels were found to reach a peak after 48 h of culture

Previous reports indicated that the expression of CCL1 mRNA and protein is induced by the HTLV-1 Tax oncoprotein [17, 24]. However, these data were obtained by northern blotting, PCR, and ELISA analyses using whole cells. In this study, we confirmed these previous findings and further extended the analysis to the single-cell level using flow cytometry. We used JPX9 cells [20], a Jurkat (HTLV-1-negative, human T-cell leukemia cell line) subclone generated by stable transfection of a functional Tax expression-plasmid vector, and induced Tax expression by adding cadmium chloride (CdCl_2_) to the culture medium (10 μM final concentration). As shown in Fig. 2, the treatment of JPX-9 cells with CdCl_2_ induced the expression of Tax at the RNA and protein levels (Fig. 2a and b, respectively). Results of the flow cytometry analysis indicated that CCL1 was expressed exclusively in cells that also expressed Tax (Fig. 2c). ELISA analysis of the culture supernatants showed that the addition of CdCl_2_ to the culture medium resulted in a concomitant increase in CCL1 expression within 24 h with levels reaching a peak after 48 h in culture (Fig. 2d).

### Higher expression of CCR8 in tax-positive JPX9 T-cells and PBMCs from HAM/TSP patients

**Fig. 3:**
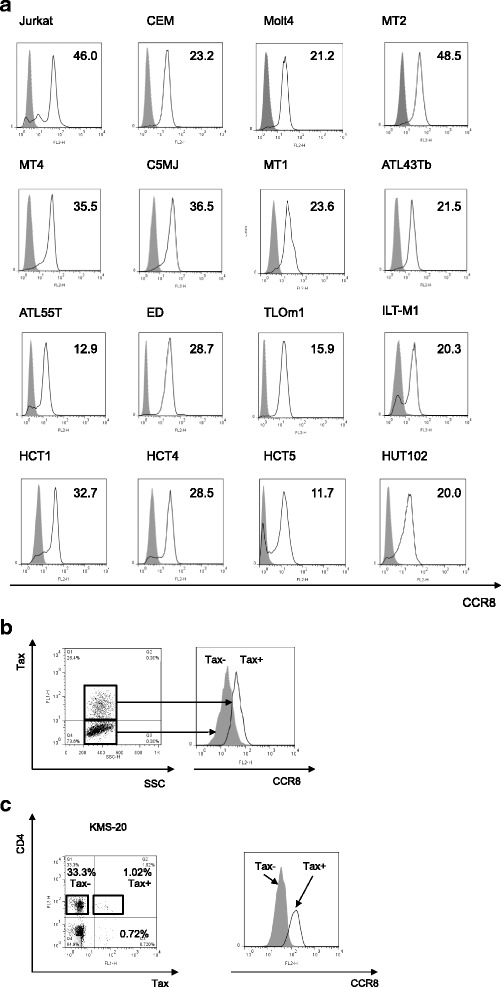
Expression of CCR8 in JPX9 T-cells and peripheral blood mononuclear cells (PBMCs) from HTLV-1-associated myelopathy/tropical spastic paraparesis (HAM/TSP) patients. The levels of CCR8 expression in HTLV-1-infected and -uninfected human T-cell lines as well as in PBMCs derived from HAM/TSP patients were examined using flow cytometry. **a**. CCR8 was expressed in both HTLV-1-infected and -uninfected T-cell lines, but the mean fluorescence intensities (MFIs) of these T-cell lines were not associated with the status of HTLV-1 infection. **b**. The MFI of CCR8 was higher in the Tax-positive fraction compared to the Tax-negative fraction in Tax-induced JPX-9 cells **c**. The MFI of CCR8 was higher in Tax-positive PBMCs than Tax-negative PBMCs derived from HAM/TSP patients

Because CCR8 is the sole receptor for human CCL1, we also assessed the levels of CCR8 expression in HTLV-1-infected and -uninfected human T-cell lines as well as PBMCs derived from HAM/TSP patients using flow cytometry. The results showed that CCR8 was expressed in both HTLV-1-infected and non-infected T-cell lines, but the mean fluorescence intensities (MFIs) of these T-cell lines were not associated with the status of HTLV-1 infection (Fig. 3a). However, the MFI of CCR8 was higher in the Tax-positive fraction than that of the Tax-negative fraction in Tax-induced JPX-9 cells (Fig. 3b). Furthermore, the MFI of CCR8 was higher in Tax-positive PBMCs than in Tax-negative PBMCs derived from HAM/TSP patients (Fig. 3c).

### HTLV-1 tax transactivates the CCL1 gene by acting concurrently on AP-1- and CREB-binding sites

**Fig. 4:**
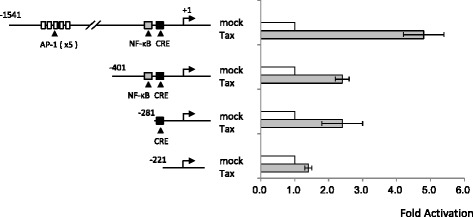
HTLV-1 Tax transactivates the *CCL1* gene by acting concurrently on AP-1- and CREB-binding sites. The luciferase gene was used to monitor the activity of the *CCL1* gene promoter. Four fragments of the 5′-flanking regulatory region of the *CCL1* gene were cloned upstream of the luciferase gene, yielding four constructs. These reporter constructs were independently transfected into Jurkat human T-cells with or without the Tax expression plasmid. The results showed that both AP-1- and CREB-binding sites were essential and sufficient for transactivation of the *CCL1* gene by HTLV-1 Tax

To examine whether the 5′-flanking sequence of the human *CCL1* gene is responsible for the expression stimulated by Tax, the *Firefly* luciferase gene was used to monitor the activity of the *CCL1* gene promoter (Fig. 4). For this purpose, we constructed four plasmids in which portions of the 5′-flanking region of *CCL1* had been deleted and cloned upstream of the luciferase gene. The reporter constructs were independently transfected into Jurkat human T-cells with or without the Tax expression plasmid. The results suggest that both the activator protein-1 (AP-1)- and the cAMP response element-binding protein (CREB)-binding sites were essential and sufficient for *CCL1* transactivation by HTLV-1 Tax.

### CCL1 plasma levels were significantly higher in patients with HAM/TSP than in HTLV-1-seronegative patients with multiple sclerosis and asymptomatic HTLV-1 healthy carriers

**Fig. 5:**
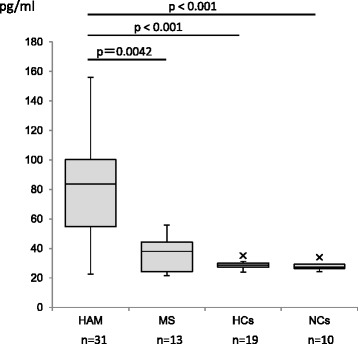
Plasma levels of CCL1 were significantly higher in patients with HTLV-1-associated myelopathy/tropical spastic paraparesis (HAM/TSP) than in HTLV-1-seronegative patients with multiple sclerosis (MS) and asymptomatic HTLV-1 healthy carriers (HC). The plasma levels of CCL1 in 31 patients with HAM/TSP, 13 HTLV-1-seronegative patients with MS, 19 HCs, and 10 normal controls (NCs) were examined by ELISA. As shown, the plasma levels of CCL1 were significantly higher in patients with HAM/TSP (mean ± SD: 79.3 ± 30.5 pg/ml) than in HTLV-1-seronegative patients with MS (36.8 ± 11.3 pg/ml), HCs (28.7 ± 2.5 pg/ml), and NCs (28.4 ± 3.2 pg/ml)

To investigate whether CCL1 expression is associated with the in vivo pathogenesis of HAM/TSP, we performed ELISAs to measure the plasma levels of CCL1 in 31 patients with HAM/TSP, 13 HTLV-1-seronegative patients with MS, 19 HCs, and 10 NCs (Fig. 5). We observed that the CCL1 plasma levels were significantly higher in patients with HAM/TSP (mean ± SD: 79.3 ± 30.5 pg/ml) than in HTLV-1-seronegative patients with MS (36.8 ± 11.3 pg/ml), HCs (28.7 ± 2.5 pg/ml), and NCs (28.4 ± 3.2 pg/ml). However, there were no correlations between serum CCL1 levels and HTLV-1 PVL or *tax* mRNA expression in PBMCs (*p* = 0.87, *r* = 0.10 and *p* = −0.21, *r* = 0.74, respectively, by Spearman’s rank correlation analysis). Also, there was no statistical correlation between plasma CCL1 levels and the disease severity (i.e.*,* OMDS) of HAM/TSP patients.

### Minocycline inhibits the production of CCL1 in HTLV-1-infected T-cell lines

**Fig. 6:**
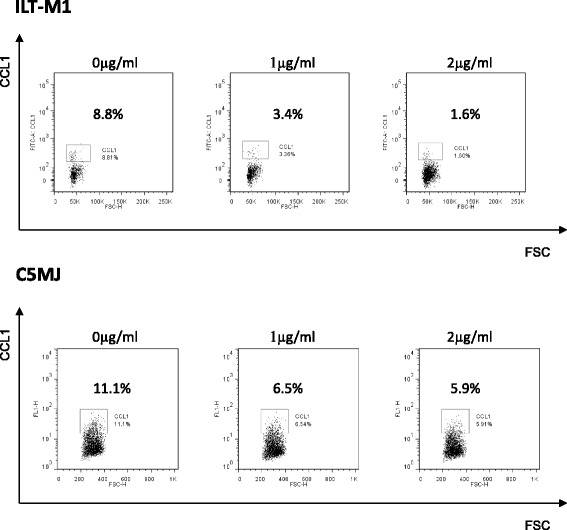
Minocycline inhibits the production of CCL1 in HTLV-1-infected T-cell lines. Effect of minocycline on CCL1 protein expression in the HTLV-1-associated myelopathy/tropical spastic paraparesis (HAM/TSP)-derived HTLV-1-infected T-cell line ILT-M1 and in the HTLV-1-transformed cell line C5MJ. Minocycline suppressed the expression of CCL1 in both ILT-M1 and C5MJ cell lines in a dose-dependent manner

It has been reported that minocycline, a widely used tetracycline antibiotic, has neuroprotective properties against excitotoxicity in several experimental models of neurological diseases by inhibiting the activation and proliferation of microglia [25, 26]. The bioavailability of minocycline is very high in humans. It is very well absorbed by the gastrointestinal tract after oral administration, and the maximum serum concentration (1–2 μg/ml) is attained within 1–4 h after oral administration of 100-mg oral doses administered twice daily. This is the concentration we used for the in vitro experiments in this study, and approximately one quarter of this concentration is equivalent to the minocycline levels observed in the CSF [27]. To test the potential therapeutic disease severity against HAM/TSP, we studied the effect of minocycline on CCL1 protein expression in the HAM/TSP-derived HTLV-1-infected T-cell line ILT-M1 and in the HTLV-1-transformed cell line C5MJ. As shown in Fig. 6, minocycline suppressed the expression of CCL1 in both ILT-M1 and C5MJ cell lines in a dose-dependent manner, suggesting a potential therapeutic role for minocycline in HAM/TSP.

## Discussion

Although neurological symptoms of HAM/TSP are certainly progressive and can lead to a deterioration in the quality of life, no ideal therapeutic strategy has been shown to significantly modify the long-term disability associated with HAM/TSP. It is therefore particularly important to develop an effective therapy to improve the long-term prognosis of HAM/TSP. Previous reports have indicated that CCL1 is one of the specific chemoattractants that induce cytokine secretion and direct the trafficking of immune cells that are more likely to cross the blood-brain barrier, enter the CNS, and attract other cells including pro-inflammatory virus-specific CD8+ cells, resulting in bystander damage of CNS tissue [28]. Meanwhile, it has also been reported that, apart from its non-antibiotic effects, the widely used broad-spectrum tetracycline antibiotic minocycline possesses anti-inflammatory properties [25, 26] by reducing leucocyte chemotaxis [29]. Most importantly, minocycline can be administered orally, is rapidly absorbed, and can cross the blood-brain barrier [28]. Recent experimental data has demonstrated the beneficial properties of minocycline in several models of acute and chronic neurodegeneration, such as ischemia [30], trauma [31, 32], and Parkinson’s disease [33]. In particular in spinal cord injuries, anti-inflammatory treatment with minocycline reduced microglial activation and lesion size after injury and also contributed to functional recovery [34]. These findings prompted us to investigate the role of CCL1 in the pathogenesis of HAM/TSP and the potential of minocycline in blocking CCL1 expression by cell lines derived from HAM/TSP patients.

Our data showed that CCL1 was preferentially expressed in HTLV-1-infected T-cell lines derived from patients with HAM/TSP, whereas its sole receptor CCR8 was broadly expressed in both HTLV-1-infected and non-infected T-cell lines. Interestingly, high levels of CCL1 were detected in the culture supernatants of HTLV-1-infected T-cell lines derived from patients with HAM/TSP (3 out of 4 tested), whereas it was not detectable in any of the other cell lines tested except for the HTLV-1-transformed C5MJ cell line. Experiments using JPX-9 cells, which is a subline of the Jurkat HTLV-1-negative human T-cell leukemia cell line that expresses biologically active Tax protein under the control of the metallothionein promoter, showed that CCL1 was expressed exclusively in Tax-positive cells, suggesting that CCL1 expression was driven exclusively by HTLV-1 Tax at the single-cell level likely because of the Tax transactivation function. Furthermore, when HTLV-1-uninfected cell lines (Jurkat, CEM and Molt4) were incubated with 10 U/ml of IL-2, the levels of CCL1 expression were unaffected. Thus, a Tax-dependent mechanism seems more likely. Indeed, our reporter gene assays showed that Tax transactivates the *CCL1* gene promoter by acting concurrently on AP-1- and on CREB-binding sites. More importantly, we also found that CCL1 plasma levels were significantly higher in patients with HAM/TSP than in HTLV-1-seronegative patients with MS and HCs, and CCR8 was expressed at higher levels in Tax-positive PBMCs than those of Tax-negative PBMCs derived from HAM/TSP patients. It is noteworthy that there was no correlation between serum CCL1 levels and HTLV-1 PVL or tax mRNA expression in PBMCs, and there was no statistical significance between plasma CCL1 levels and the disease severity (i.e., OMDS) of HAM/TSP patients, suggesting that the plasma CCL1 levels of HAM/TSP patients were not reflected by changes in levels of HTLV-1 PVL or tax mRNA expression in the systemic circulation. This is probably because other factors may also be affecting the increased plasma levels of CCL1 and clinical manifestations of HAM/TSP patients, or systemic circulation does not reflect the tissue microenvironment at the spinal cord. Nonetheless, our data suggest that the CCL1-CCR8 axis may play a role in the pathogenesis of HAM/TSP. Indeed, Enose-Akahata et al. (2012) have reported that minocycline directly inhibited activated mononuclear phagocytes (MPs) and that the downregulation of MP function can modulate CD8+ T cells (i.e., CTL) function in HAM/TSP patients [35]. Because the CTL response to HTLV-1 was robust in patients with HAM/TSP, and activated HTLV-1-specific CTLs might contribute to the tissue damage seen in HAM/TSP patients, they suggested that the inhibition of HTLV-1-infected or activated MPs by minocycline may be of clinical use in the treatment of patients with HAM/TSP. Therefore, minocycline may be potentially used as a therapeutic for HAM/TSP. Furthermore, because minocycline directly inhibits the main characteristics of HTLV-1-infected T-cells in HAM/TSP patients, it can be expected that the inhibition of CCL1 overexpression will help to relieve the main HAM/TSP symptoms.

There is growing evidence that certain antibiotics exert their clinically beneficial effects not only by killing or inhibiting the growth of bacterial pathogens, but also by their anti-inflammatory or immunomodulatory activities [36]. For instance, it is well known that the immunomodulatory properties of macrolide antibiotics have been used to successfully treat several chronic inflammatory lung disorders and improve the long-term outcome of patients with pulmonary disease [37]. Minocycline has a variety of biological properties that are independent of its anti-microbial activity, among which are the anti-inflammatory activity [25, 26] and its ease in crossing the blood–brain barrier [38] because of its high lipid solubility [39]. Thus, the anti-inflammatory or immunomodulatory effects of minocycline may set this antibiotic as an attractive therapeutic option for patients with HAM/TSP. From this point of view, one of the most interesting findings of this study is that minocycline suppressed the expression of CCL1 in HAM/TSP-derived cell lines in a dose-dependent manner. This result may suggest that CCL1 is a key molecule in the pathogenesis of HAM/TSP, and that minocycline has a potential therapeutic role in limiting inflammation in patients with HAM/TSP. Needless to say, it is important to know the side effects of minocycline such as vestibular disturbance, candida infection, gastrointestinal disturbance, cutaneous symptoms (pigmentation, pruritus, photosensitive rash, and urticaria), and benign intracranial hypertension. When such side effects occur, doctors should respond to them quickly in order to prevent more serious problems.

## Conclusion

In conclusion, we demonstrated that CCL1 was preferentially expressed in HTLV-1-infected T-cell lines derived from patients with HAM/TSP. Higher CCL1 plasma levels were found in patients with HAM/TSP in comparison with HCs and HTLV-1-uninfected patients with MS. Moreover, minocycline inhibited the production of CCL1 in a dose-dependent manner in HTLV-1-infected T-cell lines. Taken together, our results show that CCL1 may be a key molecule in the pathogenesis of HAM/TSP as well as a potential target for immunotherapy. Therefore, further analyses must be performed in CSF samples to confirm the significance of minocycline.

## Abbreviations

ATL: Adult T-cell leukemia
HAM/TSP: HTLV-1-associated myelopathy/tropical spastic paraparesis
HCs: Asymptomatic HTLV-1 healthy carriers
HTLV-1: Human T-cell leukemia virus type-1
MS: Multiple sclerosis
NCs: Normal uninfected healthy controls
